# High Mammographic Density in Younger Women and Its Implications for Breast Cancer Subtypes

**DOI:** 10.1155/ijbc/8401844

**Published:** 2026-06-29

**Authors:** Sariningsih Hikmawati, Kardinah, Rizky Ifandriani Putri, Vincent Laiman, Intan Mupangat, Rozan Muhammad Irfan, Zannuba Arifah Noor, Putri Noviantika Laskarsantri, Laksmitha Saktiono Safitri, Artanto Wahyono, Didik Setyo Heriyanto, Lina Choridah

**Affiliations:** ^1^ Doctoral Program of Medical and Health Science, Faculty of Medicine, Public Health and Nursing, Universitas Gadjah Mada, Yogyakarta, Indonesia, ugm.ac.id; ^2^ Department of Radiology, Dharmais Cancer Hospital, Jakarta, Indonesia, dharmais.co.id; ^3^ Department of Anatomical Pathology, Dharmais Cancer Hospital, Jakarta, Indonesia, dharmais.co.id; ^4^ Department of Radiology, Faculty of Medicine, Public Health and Nursing, Universitas Gadjah Mada, Yogyakarta, Indonesia, ugm.ac.id; ^5^ Dr Sardjito General Hospital, Yogyakarta, Indonesia; ^6^ Faculty of Medicine, Universitas Muhammadiyah Malang, Malang, Indonesia, umm.ac.id; ^7^ Department of Surgery, Faculty of Medicine, Public Health and Nursing, Universitas Gadjah Mada, Yogyakarta, Indonesia, ugm.ac.id; ^8^ Department of Anatomical Pathology, Faculty of Medicine, Public Health and Nursing, Universitas Gadjah Mada, Yogyakarta, Indonesia, ugm.ac.id

**Keywords:** age factors, breast density, HER2, Ki-67, mammography

## Abstract

**Background:**

High breast density is commonly observed in younger breast cancer patients (<45 years), and Asian women generally have dense breasts even beyond the age of 45 years. Its relationship with breast cancer subtypes and its profile remains unclear. This study is aimed at exploring the association between mammographic density and breast cancer molecular subtypes.

**Methods:**

A cross‐sectional study evaluating mammographic density based on the 2013 BI‐RADS classification among breast cancer patients at Dharmais Cancer Hospital between 2021 and 2023 was conducted. Clinicopathological variables—including histological subtype, tumor grade, ER, PR, HER2 status, Ki‐67 index, and molecular subtypes by immunohistochemistry—were analyzed.

**Results:**

High breast density (BI‐RADS C and D) was observed in 69% of patients, with Category C being the most common (57.1%). Invasive ductal carcinoma of no special type was the most prevalent and 47.2% of tumors were high grade. Most tumors were ER‐negative (67.1%), PR‐negative (59.6%), and HER2 0 (54.0%), with high Ki‐67 index in 70.2% of cases. Age was associated with breast Density C and D and TNBC molecular subtype (*p* < 0.05). HER2, Ki‐67, and molecular subtype were negatively correlated with ER (*r* = −0.217, *r* = −0.283, and *r* = −0.851, respectively; *p* < 0.05) and with PR (*r* = −0.169, *r* = −0.287, and *r* = −0.840, respectively; *p* < 0.05). Conversely, HER2 and Ki‐67 expressions were positively correlated with more aggressive molecular subtypes (*r* = 0.307 and *r* = 0.354, respectively; *p* < 0.05).

**Conclusion:**

Younger breast cancer patients were associated with higher breast density and more aggressive tumor subtypes, which correlated with HER2 and Ki‐67 expressions in breast cancer. These underscore the relevance of age‐related biological factors in shaping breast cancer characteristics. However, among Indonesian women over 45 years old, the proportion of high breast density remains remarkably high (65.6%), suggesting that factors beyond age—such as genetic, hormonal, or lifestyle influences—may contribute to the persistence of dense breast tissue in this population.

## 1. Introduction

Breast cancer is a significant global health concern and remains the leading cause of cancer‐related mortality among women worldwide, including in Indonesia [1]. Annually, approximately 2.3 million new cases are diagnosed globally, contributing to 11.7% of all new cancer cases, with a staggering mortality rate of 685,000 (6.9%). In Indonesia, the situation is particularly alarming, with an estimated 65,858 new cases (16.6%) and 22,430 deaths (9.6%) each year, reflecting a higher mortality rate compared to the global average [1].

Mammography is the gold standard for breast cancer screening, effectively reducing the stage at diagnosis and improving treatment outcomes [2]. However, high mammographic density can significantly compromise the sensitivity of mammographic examinations, making it challenging to detect tumors in dense breast tissue [2]. Mammography density is defined as the proportion of fibroglandular tissue relative to fatty tissue in the breast, and it varies among individuals and can change over a woman′s lifetime. Factors such as age, menopausal status, body mass index, hormone replacement therapy, and parity have all been linked to variations in mammographic density, which are also recognized as risk factors for breast cancer [3].

Research has established a positive correlation between mammographic density and the risk of invasive breast tumors across all age groups, with the highest density levels significantly increasing risk. Notably, studies have shown a stronger association between high mammographic density and aggressive tumor subtypes, such as triple‐negative breast cancer (TNBC) and estrogen receptor (ER)‐negative tumors, particularly in younger women [4, 5]. However, investigations specifically examining the relationship between mammographic density and molecular subtypes of breast cancer remain limited, particularly in Indonesia.

HER2 is one of the routinely examined molecular markers in breast cancer and plays a critical role in tumor classification [6]. According to the latest American Society of Clinical Oncology/College of American Pathologists (ASCO/CAP) guidelines, tumors with a human epidermal growth factor receptor 2 (HER2) immunohistochemistry (IHC) score of 1+, previously considered HER2‐negative, are now categorized as HER2‐low [6]. Furthermore, the novel antibody‐drug conjugate (ADC) trastuzumab deruxtecan has shown promising therapeutic benefit in patients with advanced or metastatic HER2‐low breast cancer who had previously received treatment, as evidenced by the phase III DESTINY‐Breast04 trial [7]. These have prompted the consideration of HER2‐low tumors as a distinct biological subtype of breast cancer. Nevertheless, information on the association of HER2‐low status and breast density remains limited.

This study is aimed at filling this critical gap by evaluating mammographic density and exploring its association with breast cancer molecular subtypes in Indonesia, as well as the new HER2 classification. By enhancing our understanding of these relationships, this research seeks to contribute to improved screening, diagnosis, and treatment strategies for breast cancer, ultimately leading to better patient outcomes.

## 2. Methods

### 2.1. Study Design

This research was designed as a cross‐sectional study to evaluate mammographic density using visual assessment agreement of two breast radiology consultants with experience more than 10 years based on Breast Imaging Reporting and Data System (BI‐RADS) density categories, alongside the determination of molecular subtypes of breast cancer in the study subjects. Data were analyzed to explore relationships among various variables. Data were collected retrospectively using Digital Imaging and Communications in Medicine (DICOM) format. The target population comprised breast cancer patients at Dharmais Cancer Hospital since 2021, all of whom had been definitively diagnosed through histopathological examination.

### 2.2. Patients Characteristics

Inclusion criteria for the study included female breast cancer patients with confirmed histopathological diagnoses, available mammograms, and complete IHC profiles including molecular subtype data. Exclusion criteria encompassed patients without complete medical records, those with poor‐quality mammographic images. Sampling was conducted through consecutive sampling, which is appropriate for clinical research involving newly diagnosed cases.

The study was conducted at the Faculty of Medicine, Public Health, and Nursing, Universitas Gadjah Mada, and was approved by the Ethics Committee (approval number: KE/FK/0471/EC/2025). All patient data were anonymized in accordance with ethical guidelines.

### 2.3. Data Collection

The mammographic density assessment was determined by consensus between two board‐certified breast radiology consultants. Breast composition was categorized according to BI‐RADS criteria A, B, C, and D and also categorized as high density (BI‐RADS C and D) and low density (BI‐RADS A and B). Histological grade, ER, progesterone receptor (PR), HER2, Ki‐67, and molecular subtype results were obtained from patient medical records. Patients with equivocal HER2 scores (2+) underwent further testing using the HercepTest to determine whether the result should be classified as HER2‐low (1+) or HER2‐positive (3+). Statistical analyses included chi‐square tests, *t*‐tests, and Pearson correlation. A *p* value < 0.05 was considered statistically significant.

## 3. Results

We included 161 patients, with a mean age of 53.37 ± 9.80 years, ranging from 32 to 85 years (Table 1). A total of 6.7% of participants were classified as young breast cancer patients, defined as being under 45 years of age. Approximately 69% of patients had high breast density (BI‐RADS Categories C and D), with heterogeneously dense breasts (Category C) being the most common (57.1%). In the age group under 45 years, high breast density was observed in 83.3% of women, whereas in those over 45 years, it was 65.6%. The remaining 31% had low breast density (Categories A and B). We observed that 67.1% of patients were ER negative and 59.6% were PR negative. Additionally, more than half (54.0%) were HER2 + 0, and a high Ki‐67 level was found in 70.2% of patients.

**Table 1:** Subject characteristics.

Characteristics	Statistics *N* = 161 (100%)
Age	
< 45 years (young breast cancer)	30 (6.7)
> 45 years	131 (93.3)
Mean (SD)	53.37 (9.804)
Median (range)	52 (32‐85)

Mammography density	
A: the breasts are almost entirely fatty	13 (8.1)
B: there are scattered areas of fibroglandular density	37 (23)
C: the breasts are heterogeneously dense, which may obscure small masses	92 (57.1)
D: the breasts are extremely dense, which lowers the sensitivity of mammography	19 (11.8)
Low (A + B)	50 (31)
High (C + D)	111 (69)

ER	
Positive	53 (32.9)
Negative	108 (67.1)

PR	
Positive	65 (40.4)
Negative	96 (59.6)

HER2	
HER2 + 0	87 (54.0)
HER2 + 1	33 (20.5)
HER2 + 3	41 (25.5)

Ki‐67	
Low	48 (29.8)
High	113 (70.2)

Histology findings	
DCIS	7 (4.3)
Invasive carcinoma of NST	121 (75.2)
Invasive carcinoma of NST mixed (DCIS, mucinous, lobular, metaplastic carcinoma)	11 (6.8)

Special type:	
Invasive lobular carcinoma	10 (6.2)
Mucinous carcinoma	6 (3.7)
Invasive papillary carcinoma	5 (3.1)
Metaplastic carcinoma	1 (0.6)

Histological grade	
Grade 1	14 (8.7)
Grade 2	71 (44.1)
Grade 3	76 (47.2)

Abbreviations: DCIS, ductal carcinoma in situ; ER, estrogen receptor; NST, no special type; PR, progesterone receptor.

Histopathological examination showed that the majority of tumors (70.2%) were invasive ductal carcinoma of no special type (NST). Other histologic types included invasive lobular carcinoma (5.6%), ductal carcinoma in situ (DCIS) (3.7%), mucinous carcinoma (3.1%), and several rare or mixed forms of breast cancer. We observed that 47.2% of the tumors were classified as Grade 3 tumors, whereas Grade 2 tumors accounted for 44.1% of cases, and Grade 1 tumors were observed in 8.7% of patients.

We observed significant association between age and mammographic density (Table 2) (*p* = 0.005). Among patients younger than 45 years, the majority (53.3%) fell into Category C, followed by 30.0% in Category D, 13.3% in category B, and only 3.3% in Category A. In contrast, patients aged 45 years and older were most frequently classified in Category C (58.0%) and Category B (25.2%), whereas only 10.7% were in Category D and 9.2% in Category A. However, no statistically significant association was observed between ER, PR, HER2, Ki‐67 status, molecular subtypes, or histological grade and breast density.

**Table 2:** Association between mammography density with age, histology grades, hormonal receptors, HER2, Ki‐67, and molecular subtypes.

Variables	Mammography density (*N* = 161)				*p*
	A (*n* = 13)	B (*n* = 37)	C (*n* = 92)	D (*n* = 19)	
**Age**					
< 45 years	1 (3.3)	4 (13.3)	16 (53.3)	9 (30.0)	0.005 ^∗^
≥ 45 years	12 (9.2)	33 (25.2)	76 (58.0)	10 (7.6)	

**Histology grades**					
Grade 1	1 (7.1)	3 (21.4)	8 (57.1)	2 (14.3)	0.915
Grade 2	7 (9.9)	14 (19.7)	40 (56.3)	10 (14.1)	
Grade 3	5 (6.6)	20 (26.3)	44 (7.9)	7 (9.2)	

**ER**					
Negative	3 (5.7)	13 (24.5)	29 (54.7)	8(15.1)	0.692
Positive	10 (9.3)	24 (22.2)	63 (58.3)	11 (10.2)	

**PR**					
Negative	3 (4.6)	15 (23.1)	40 (61.5)	7 (10.8)	0.555
Positive	10 (10.4)	22 (22.9)	52 (54.2)	12 (12.5)	

**HER2**					
HER2 + 0	10 (11.5)	19 (21.8)	50 (57.5)	8 (9.2)	0.328
HER2 + 1	0 (0.0)	10 (30.3)	17 (51.5)	6 (18.2)	
HER2 + 3	3 (7.3)	8 (19.5)	25 (61.0)	5 (12.2)	

**Ki-67**					
Low	3 (6.3)	13 (27.1)	24 (50.0)	8 (16.7)	0.429
High	10 (8.8)	24 (21.2)	68 (60.2)	11 (9.7)	

**Molecular subtypes**					
Luminal A	7 (9.5)	18 (24.3)	42 (56.8)	7 (9.5)	0.772
Luminal B	3 (8.1)	6 (16.2)	22 (59.5)	6 (16.2)	
HER2	0 (0)	5 (22.7)	15 (68.2)	2 (9.1)	
TNBC	3 (10.7)	8 (28.6)	13 (46.4)	4 (14.3)	

*Note:*  ^∗^statistically significant association.

Abbreviations: ER, estrogen receptor; PR, progesterone receptor.

Table 3 presents the relationship between patient age and various breast cancer biomarkers and classifications. We found a significant association between age and molecular subtype (*p* = 0.027). Luminal A was the most common subtype, particularly among older patients (48.1% vs. 36.7%), whereas Luminal B was more frequent in younger patients (26.7% vs. 22.1%). However, no significant associations were observed between age classification (< 45 vs. ≥ 45 years) and ER, PR, HER2, Ki‐67 status, or histological grade.

**Table 3:** Association between age classification with histological grade, ER, PR, HER2, Ki‐67, and molecular subtype.

Variables	Age		*p*
	< 45 years (%) (*n* = 30)	≥ 45 years (%) (*n* = 131)	
**Histology grades**			
Grade 1	2 (6.7)	12 (9.2)	0.145
Grade 2	9 (30.0)	62 (47.3)	
Grade 3	19 (63.3)	57 (43.5)	

**ER**			
Negative	13 (43.3)	40 (30.5)	0.178
Positive	17 (56.7)	91 (69.5)	

**PR**			
Negative	13 (43.3)	52 (39.7)	0.714
Positive	17 (56.7)	79 (60.3)	

**HER2**			
HER2 + 0	16 (53.5)	71 (54.2)	0.571
HER2 + 1	8 (26.7)	25 (19.1)	
HER2 + 3	6 (20.0)	35 (26.7)	

**Ki-67**			
Low	7 (23.3)	41 (31.3)	0.390
High	23 (76.7)	90 (68.7)	

**Molecular subtypes**			
Luminal A	11 (36.7)	63 (48.1)	0.027 ^∗^
Luminal B	8 (26.7)	29 (22.1)	
HER2	1 (3.3)	21 (16.0)	
TNBC	10 (33.3)	18 (13.7)	

Abbreviations: ER, estrogen receptor; PR, progesterone receptor.

Further correlation analysis showed that age was negatively correlated with breast density (*r* = −0.328, *p* < 0.05) (Table 4). Histological grade was negatively correlated with ER and PR (*r* = −0.234 and *r* = −0.267, respectively) and positively correlated with Ki‐67 and molecular subtype (*r* = 0.373 and *r* = 0.273, respectively; *p* < 0.05). ER was positively correlated with PR (*r* = 0.797; *p* < 0.05) and negatively correlated with HER2, Ki‐67, and molecular subtype (*r* = −0.217, *r* = −0.283, and *r* = −0.851, respectively; *p* < 0.05). PR was also negatively correlated with HER2, Ki‐67, and molecular subtype (*r* = −0.169, *r* = −0.287, and *r* = −0.840, respectively; *p* < 0.05). HER2 showed a positive correlation with Ki‐67 (*r* = 0.185; *p* < 0.05), and the molecular subtype was positively correlated with both HER2 and Ki‐67 (*r* = 0.307 and *r* = 0.354, respectively; *p* < 0.05).

**Table 4:** Correlation analysis table.

	Age	Breast density	Histology grade	ER	PR	HER2	Ki‐67
Age							
Breast Density	−0.328 ^∗^						
Histology Grade	−0.124	−0.052					
ER	0.095	−0.05	−0.234 ^∗^				
PR	0.001	−0.049	−0.267 ^∗^	0.797 ^∗^			
HER2	−0.092	0.087	−0.02	−0.217 ^∗^	−0.169 ^∗^		
Ki‐67	−0.098	−0.026	0.373 ^∗^	−0.283 ^∗^	−0.287 ^∗^	0.185 ^∗^	
Molecular subtype	−0.154	0.037	0.273 ^∗^	−0.851 ^∗^	−0.84 ^∗^	0.307 ^∗^	0.354 ^∗^

Abbreviations: ER, estrogen receptor; PR, progesterone receptor.

^∗^ *p* < 0.05.

## 4. Discussion

We included 161 breast cancer patients with a mean age of 53.3 years. This finding is consistent with the average age at breast cancer diagnosis reported in Asian populations but is notably lower than that observed in Western countries, where the average age is approximately 62 years [8]. The majority of patients in our cohort presented with high mammographic breast density, with BI‐RADS Category C being the most common. This observation aligns with previous research among Taiwanese women, which reported that over 80% of women under 55 years had mammograms classified as extremely or heterogeneously dense. Although the prevalence of dense breasts remained high, it decreased to 59.3% among women aged 60–64 years [9].

We observed a significant association between age and breast density. Among patients younger than 45 years, the most common breast density categories were C and D, whereas in patients aged 45 years and older, Categories C and B were the most prevalent. These findings were also supported by several previous studies. For instance, a study in China reported that 83% of women aged 45–49 had dense breasts, with the prevalence decreasing to 33.7% among women in their 60s [10]. Similarly, another study from New York found that 74% of women aged 40–49 had dense breasts, with the proportion declining to 44% among women in their 60s and 36% in their 70s [11]. These findings suggest that younger women tend to have higher mammographic density, largely due to physiological changes in which glandular tissue is progressively replaced by fatty tissue as part of the natural aging process [12]. However, their association with breast cancer remains unclear. Overall, in this study, the proportion of women over 45 years old with high breast density was also very high, reinforcing the theory that breast density is a strong risk factor for breast cancer. Further research comparing breast density between breast cancer patients and healthy individuals is needed to evaluate the extent to which breast density contributes as a risk factor for breast cancer.

We also observed a significant association between age and molecular subtype. Among women aged < 45 years, the most common subtypes were Luminal A and TNBC. In contrast, among women aged ≥45 years, Luminals A and B were the predominant subtypes. A previous large‐scale study among non‐Hispanic Asian women also demonstrated that the incidence of Luminal A breast cancer increased with age, particularly among those aged 40–69 years [13]. In that study, the prevalence of Luminal A was highest in women aged > 45 years (72.7%), followed by Luminal B (11.9%). Among women aged < 45 years, Luminal A was still the most common (59.9%), followed by Luminal B (18.6%) and TNBC (13.8%) [13]. Together, these findings suggest that older women are more likely to develop Luminals A and B tumors, subtypes associated with a better prognosis compared to more aggressive subtypes such as TNBC [14, 15].

**Figure 1:**
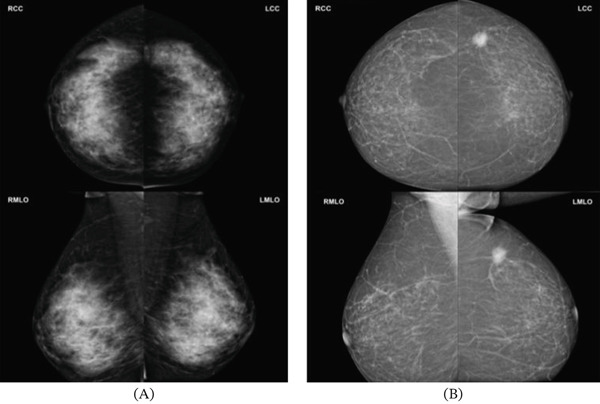
Representative mammograms showing (A) a younger patient with high breast density and (B) an older patient with low breast density.

**Figure 2:**
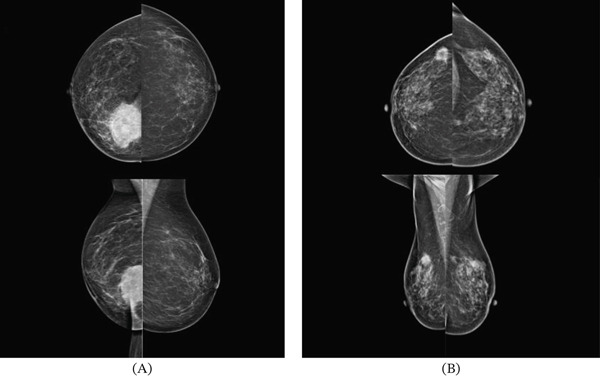
Representative mammograms of (A) triple‐negative breast cancer in a younger patient and (B) Luminal A breast cancer in an older patient.

**Figure 3:**
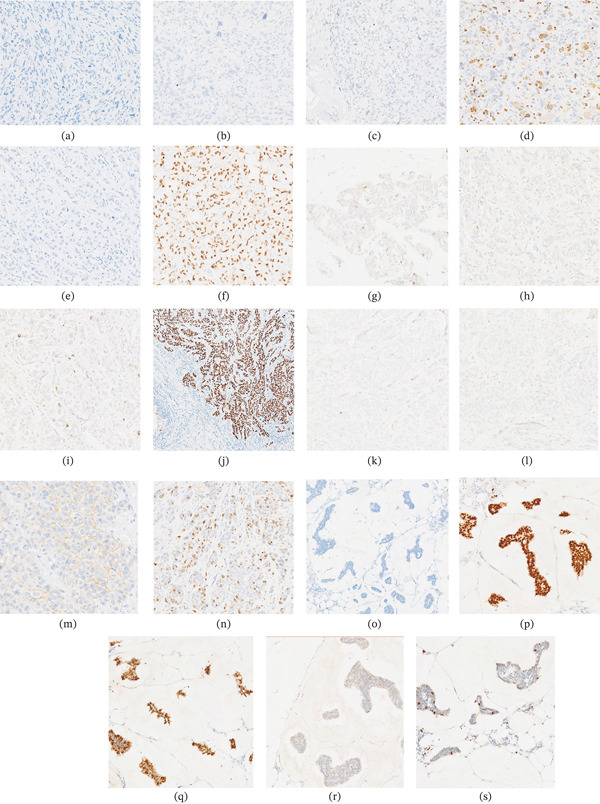
Representative immunohistochemical staining of four breast cancer cases. Patient 1, (a–d) triple‐negative with high Ki‐67 (~70%). Patient 2, (e–i) Luminal A with high ER (~80%), low PR (~10%), and low Ki‐67 (< 20%). Patient 3, (j–n) HER2‐enriched with equivocal HER2 and intermediate Ki‐67 (~40%). Patient 4, (o–s) Luminal A with strong ER/PR (~95%) and low Ki‐67 (< 5%). All images at ×100 magnification.

To understand the contribution of tumor protein profiles in breast cancer, a correlation analysis was conducted. First, we observed a negative correlation between breast density and age. A previous study in Malaysia also showed that increasing age is significantly associated with lower mammographic density [16]. This further supports our previous finding that younger patients tend to have higher breast density, particularly in Categories C and D (Figure 1). We then found that ER and PR expression were negatively correlated with histological grade, HER2 status, Ki‐67 expression, and molecular subtype, consistent with findings from previous studies. Bothou et al. [17] reported that higher tumor grade was significantly associated with reduced ER and PR expression which suggest that high‐grade tumors are more likely to be hormone receptor‐negative. Additionally, ER and PR were previously shown to be positively correlated with one another, although both exhibited inverse relationships with HER2 expression [18].The determination of hormone receptor status is a critical predictive factor in breast cancer treatment. Although HER2 overexpression is commonly associated with higher‐grade tumors, ER and PR positivity are generally linked to lower‐grade tumors, which may reflect better responsiveness to hormone therapy and a more favorable prognosis [18]. Additionally, those with more aggressive subtype such as TNBC commonly presents as a mass and is less often associated with focal asymmetry, microcalcifications, or architectural distortion (Figure 2) [19]. These masses are typically round, oval, or lobular with circumscribed, microlobulated, or obscured margins, whereas spiculated margins are rare. In contrast, non‐TNBC tumors more frequently display spiculated and indistinct margins. Notably, we observed that HER2 expression was positively correlated with both Ki‐67 expression and molecular subtype. This finding is consistent with several studies that have confirmed the association between HER2 positivity, elevated Ki‐67 levels, and poor prognostic features [20–22]. Our findings also support this relationship, as Ki‐67 was positively correlated with both histological grade and molecular subtype. Tumors with a high Ki‐67 proliferation index (≥ 15%) were associated with higher histological grades and more aggressive subtypes [20, 23]. Together, these suggest that patient age, breast density, hormone receptor status, HER2 expression, and Ki‐67 proliferation index contribute to characteristics of breast cancer. These findings are further supported by the representative immunohistochemical patterns illustrated in (Figure 3), which demonstrate the heterogeneity of molecular subtypes through distinct expression profiles of ER, PR, HER2, p53, and Ki‐67 across different patients. Beyond the classical HER2 classification, recent updates to the ASCO/CAP guidelines have introduced a more nuanced approach to HER2 status by distinguishing between HER2‐negative, HER2‐low, and HER2‐positive categories [6]. Historically, tumors with HER2 1+ scores were classified as HER2‐negative. However, emerging clinical evidence has shown that patients with HER2‐low tumors (IHC 1+ or 2+ without HER2 gene amplification) may benefit from novel targeted therapies, leading to the recognition of HER2‐low as a distinct biological subgroup [24]. This group represents a substantial proportion of breast cancers, accounting for approximately 45%–60% of cases. We observed that 20.5% of breast cancer cases in our study were classified as HER2‐low. Nevertheless, clinical trials such as DESTINY‐Breast04 have demonstrated significant improvements in progression‐free survival and overall survival among HER2‐low breast cancer patients treated with ADCs [25]. Additionally, a study from China on breast cancer patients treated with neoadjuvant chemotherapy (NACT) suggested that low HER2 expression may contribute to resistance to NACT [26]. We therefore assessed whether HER2‐low status is associated with breast density and observed a trend indicating that HER2‐low tumors were least likely found in patients with Category A breast density. This finding is consistent with a previous systematic review, which reported that women in the highest breast density category had an increased risk of developing HER2‐positive breast cancer [27]. The lack of statistically significant differences may be related to the limited sample size. Nevertheless, the relationship between breast density and HER2‐positive subtypes remains unclear. Despite this, high breast density retains clinical importance, as it may reduce mammographic sensitivity, delay detection, and necessitate supplemental imaging while also serving as an independent risk factor influencing screening strategies.

## 5. Conclusion

Our study showed that both breast density and molecular subtype were associated with age, with younger patients tending to have denser breasts and a higher proportion of more aggressive subtypes. Additionally, HER2 and Ki‐67 expressions were positively correlated with more aggressive molecular subtypes. Future studies integrating genomic investigations—such as quantitative qRT‐PCR testing of HER2 mRNA levels—are warranted to further explore the molecular mechanisms linking breast density and breast cancer subtypes, particularly in HER2‐low tumors. However, among Indonesian women over 45 years old, the proportion of high breast density remains remarkably high (65.6%).

**Nomenclature**
ADC: antibody‐drug conjugate
ASCO/CAP: American Society of Clinical Oncology/College of American Pathologists
BI‐RADS: Breast Imaging Reporting and Data System
DCIS: ductal carcinoma in situ
DICOM: Digital Imaging and Communications in Medicine
ER: estrogen receptor
HER2: human epidermal growth factor receptor 2
NACT: neoadjuvant chemotherapy
NST: no special type
PR: progesterone receptor
TNBC: triple negative breast cancer

## Author Contributions

All authors contributed substantially to the concept and design of the study, drafting of the article, and critically revising the manuscript for important intellectual content. L.C. and S.H.: conceptualization, methodology, software. R.I.P., L.S.S., and I.M.: data curation. K.: writing—original draft preparation. A.W. and D.S.H.: visualization, investigation. D.S.H., L.C., and S.H.: supervision. V. L. and R.M.I.: software, validation. P.N.L. and Z.A.N.: writing—reviewing and editing.

## Funding

This work was supported by the Dana Masyarakat Tahun 2024, Faculty of Medicine, Public Health and Nursing, Universitas Gadjah Mada, Yogyakarta, No. 1533/UN1/FKKMK/PPKE/PT/2024.

## Disclosure

All authors have read and approved the final version of the manuscript for publication.

## Ethics Statement

This study was approved by approved by The Medical and Health Research Ethics Committee of Faculty of Medicine, Public Health, and Nursing Universitas Gadjah Mada, Yogyakarta, Indonesia (approval number: KE/FK/0471/EC/2025).

## Consent

The authors have nothing to report..

## Conflicts of Interest

The authors declare that they have no conflicts of interest.

## Data Availability

The data that support the findings of this study are available from the corresponding author upon reasonable request.

## References

[1] Sung H. , Ferlay J. , Siegel R. L. , Laversanne M. , Soerjomataram I. , Jemal A. , and Bray F. , Global Cancer Statistics 2020: GLOBOCAN Estimates of Incidence and Mortality Worldwide for 36 Cancers in 185 Countries, CA: A Cancer Journal for Clinicians. (2021) 71, no. 3, 209–249, 10.3322/caac.21660, 33538338. 33538338

[2] Shi J. , Li J. , Gao Y. , Chen W. , Zhao L. , Li N. , Tian J. , and Li Z. , The Screening Value of Mammography for Breast Cancer: An Overview of 28 Systematic Reviews With Evidence Mapping, Journal of Cancer Research and Clinical Oncology. (2025) 151, no. 3, 10.1007/s00432-025-06122-z, 40047905. PMC11885354 40047905

[3] Eriksson L. , Hall P. , Czene K. , dos Santos S. I. , McCormack V. , Bergh J. , Bjohle J. , and Ploner A. , Mammographic Density and Molecular Subtypes of Breast Cancer, British Journal of Cancer. (2012) 107, no. 1, 18–23, 10.1038/bjc.2012.234, 22644308. 22644308 PMC3389424

[4] Bertrand K. A. , Tamimi R. M. , Scott C. G. , Jensen M. R. , Pankratz V. S. , Visscher D. , Norman A. , Couch F. , Shepherd J. , Fan B. , Chen Y. Y. , Ma L. , Beck A. H. , Cummings S. R. , Kerlikowske K. , and Vachon C. M. , Mammographic Density and Risk of Breast Cancer by Age and Tumor Characteristics, Breast Cancer Research. (2013) 15, no. 6, 10.1186/bcr3570, 24188089. PMC3978749 24188089

[5] Sartor H. , Borgquist S. , Hartman L. , Olsson Å. , Jawdat F. , and Zackrisson S. , Do Mammographic Tumor Features in Breast Cancer Relate to Breast Density and Invasiveness, Tumor Size, and Axillary Lymph Node Involvement?, Acta Radiologica. (2015) 56, no. 5, 536–544, 10.1177/0284185114532081, 24814360. 24814360

[6] Wolff A. C. , Hammond M. E. H. , Allison K. H. , Harvey B. E. , Mangu P. B. , Bartlett J. M. S. , Bilous M. , Ellis I. O. , Fitzgibbons P. , Hanna W. , Jenkins R. B. , Press M. F. , Spears P. A. , Vance G. H. , Viale G. , McShane L. M. , and Dowsett M. , Human Epidermal Growth Factor Receptor 2 Testing in Breast Cancer: American Society of Clinical Oncology/College of American Pathologists Clinical Practice Guideline Focused Update, Journal of Clinical Oncology. (2018) 36, no. 20, 2105–2122, 10.1200/jco.2018.77.8738, 29846122. 29846122

[7] Modi S. , Jacot W. , Yamashita T. , Sohn J. , Vidal M. , Tokunaga E. , Tsurutani J. , Ueno N. T. , Prat A. , Chae Y. S. , Lee K. S. , Niikura N. , Park Y. H. , Xu B. , Wang X. , Gil-Gil M. , Li W. , Pierga J. Y. , Im S. A. , Moore H. C. F. , Rugo H. S. , Yerushalmi R. , Zagouri F. , Gombos A. , Kim S. B. , Liu Q. , Luo T. , Saura C. , Schmid P. , Sun T. , Gambhire D. , Yung L. , Wang Y. , Singh J. , Vitazka P. , Meinhardt G. , Harbeck N. , Cameron D. A. , and DESTINY-Breast04 Trial Investigators , Trastuzumab Deruxtecan in Previously Treated HER2-Low Advanced Breast Cancer, New England Journal of Medicine. (2022) 387, no. 1, 9–20, 10.1056/NEJMoa2203690, 35665782. 35665782 PMC10561652

[8] Giaquinto A. N. , Sung H. , Newman L. A. , Freedman R. A. , Smith R. A. , Star J. , Jemal A. , and Siegel R. L. , Breast Cancer Statistics 2024, CA: A Cancer Journal for Clinicians. (2024) 74, no. 6, 477–495, 10.3322/caac.21863. 39352042

[9] Liao Y.-S. , Zhang J.-Y. , Hsu Y.-C. , Hong M.-X. , and Lee L.-W. , Age-Specific Breast Density Changes in Taiwanese Women: A Cross-Sectional Study, International Journal of Environmental Research and Public Health. (2020) 17, no. 9, 10.3390/ijerph17093186, 32375295. PMC7246480 32375295

[10] Yan H. , Ren W. , Jia M. , Xue P. , Li Z. , Zhang S. , He L. , and Qiao Y. , Breast Cancer Risk Factors and Mammographic Density Among 12518 Average-Risk Women in Rural China, BMC Cancer. (2023) 23, no. 1, 10.1186/s12885-023-11444-7, 37814233. PMC10561452 37814233

[11] Checka C. M. , Chun J. E. , Schnabel F. R. , Lee J. , and Toth H. , The Relationship of Mammographic Density and Age: Implications for Breast Cancer Screening, American Journal of Roentgenology. (2012) 198, no. 3, W292–W295, 10.2214/ajr.10.6049, 22358028. 22358028

[12] Ohmaru A. , Maeda K. , Ono H. , Kamimura S. , Iwasaki K. , Mori K. , and Kai M. , Age-Related Change in Mammographic Breast Density of Women Without History of Breast Cancer Over a 10-Year Retrospective Study, PeerJ. (2023) 11, e14836, 10.7717/peerj.14836, 36815981. 36815981 PMC9936867

[13] Acheampong T. , Kehm R. D. , Terry M. B. , Argov E. L. , and Tehranifar P. , Incidence Trends of Breast Cancer Molecular Subtypes by Age and Race/Ethnicity in the US From 2010 to 2016, JAMA Network Open. (2020) 3, no. 8, e2013226, 10.1001/jamanetworkopen.2020.13226, 32804214. 32804214 PMC7431997

[14] Mills M. , Liveringhouse C. , Lee F. , Nanda R. H. , Ahmed K. A. , Washington I. R. , Thapa R. , Fridley B. L. , Blumencranz P. , Extermann M. , Loftus L. , Balducci L. , and Diaz R. , The Prevalence of Luminal B Subtype Is Higher in Older Postmenopausal Women With ER+/HER2-Breast Cancer and is Associated With Inferior Outcomes, Journal of Geriatric Oncology. (2021) 12, no. 2, 219–226, 10.1016/j.jgo.2020.08.007, 32859560. 32859560 PMC7907245

[15] Agresti R. , Meneghini E. , Baili P. , Minicozzi P. , Turco A. , Cavallo I. , Funaro F. , Amash H. , Berrino F. , Tagliabue E. , and Sant M. , Association of Adiposity, Dysmetabolisms, and Inflammation With Aggressive Breast Cancer Subtypes: A Cross-Sectional Study, Breast Cancer Research and Treatment. (2016) 157, no. 1, 179–189, 10.1007/s10549-016-3802-3, 27117160. 27117160

[16] Hanis T. M. , Arifin W. N. , Haron J. , Wan Abdul Rahman W. F. , Ruhaiyem N. I. R. , Abdullah R. , and Musa K. I. , Factors Influencing Mammographic Density in Asian Women: A Retrospective Cohort Study in the Northeast Region of Peninsular Malaysia, Diagnostics. (2022) 12, no. 4, 10.3390/diagnostics12040860, 35453907. PMC9032698 35453907

[17] Bothou A. , Estrogen Receptor (ER) and Progesterone Receptor (PR) Status in Breast Cancer and Its Association With Histological Grade: A Case Series Study, Journal of Clinical Images and Medical Case Reports. (2024) 5, no. 5, 3071, 10.52768/2766-7820/3071.

[18] Siadati S. , Sharbatdaran M. , Nikbakhsh N. , and Ghaemian N. , Correlation of ER, PR and HER-2/Neu With Other Prognostic Factors in Infiltrating Ductal Carcinoma of Breast, Iranian Journal of Pathology. (2015) 10, no. 3, 221–226, 26351488. 26351488 PMC4539777

[19] Gao B. , Zhang H. , Zhang S. D. , Cheng X. Y. , Zheng S. M. , Sun Y. H. , Zhang D. W. , Jiang Y. , and Tian J. W. , Mammographic and Clinicopathological Features of Triple-Negative Breast Cancer, British Journal of Radiology. (2014) 87, no. 1039, 20130496, 10.1259/bjr.20130496, 24734934. 24734934 PMC4075572

[20] Inwald E. C. , Klinkhammer-Schalke M. , Hofstädter F. , Zeman F. , Koller M. , Gerstenhauer M. , and Ortmann O. , Ki-67 Is a Prognostic Parameter in Breast Cancer Patients: Results of a Large Population-Based Cohort of a Cancer Registry, Breast Cancer Research and Treatment. (2013) 139, no. 2, 539–552, 10.1007/s10549-013-2560-8, 23674192. 23674192 PMC3669503

[21] Haroon S. , Hashmi A. A. , Khurshid A. , Kanpurwala M. A. , Mujtuba S. , Malik B. , and Faridi N. , Ki67 Index in Breast Cancer: Correlation With Other Prognostic Markers and Potential in Pakistani Patients, Asian Pacific Journal of Cancer Prevention. (2013) 14, no. 7, 4353–4358, 10.7314/apjcp.2013.14.7.4353, 23992002. 23992002

[22] Madani S.-H. , Payandeh M. , Sadeghi M. , Motamed H. , and Sadeghi E. , The Correlation Between Ki-67 With Other Prognostic Factors in Breast Cancer: A Study in Iranian Patients, Indian Journal of Medical and Paediatric Oncology. (2016) 37, no. 2, 95–99, 10.4103/0971-5851.180136, 27168707. 27168707 PMC4854054

[23] Nahed A. S. and Shaimaa M. Y. , Ki-67 as a Prognostic Marker According to Breast Cancer Molecular Subtype, Cancer Biology & Medicine. (2016) 13, no. 4, 496–504, 10.20892/j.issn.2095-3941.2016.0066, 28154782. 28154782 PMC5250608

[24] Tarantino P. , Hamilton E. , Tolaney S. M. , Cortes J. , Morganti S. , Ferraro E. , Marra A. , Viale G. , Trapani D. , Cardoso F. , Penault-Llorca F. , Viale G. , Andrè F. , and Curigliano G. , HER2-Low Breast Cancer: Pathological and Clinical Landscape, Journal of Clinical Oncology. (2020) 38, no. 17, 1951–1962, 10.1200/jco.19.02488. 32330069

[25] Nader-Marta G. , Singer C. , Hlauschek D. , DeMichele A. , Tarantino P. , de Azambuja E. , Pfeiler G. , Martin M. , Balko J. M. , Nowecki Z. , Balic M. , Brufsky A. M. , Chan A. , Morris P. G. , Haddad T. , Loibl S. , Liu Y. , Soelkner L. , Fesl C. , Mayer E. L. , Gnant M. , and on behalf of the PALLAS groups and investigators , Clinical Characterization, Prognostic, and Predictive Values of HER2-Low in Patients With Early Breast Cancer in the PALLAS Trial (ABCSG-42/AFT-05/BIG-14–13/PrE0109), Breast Cancer Research. (2024) 26, no. 1, 10.1186/s13058-024-01899-2, 39375745. PMC11459983 39375745

[26] Wang W. , Zhu T. , Chen H. , and Yao Y. , The Impact of HER2-Low Status on Response to Neoadjuvant Chemotherapy in Clinically HER2-Negative Breast Cancer, Clinical and Translational Oncology. (2023) 25, no. 6, 1673–1681, 10.1007/s12094-022-03062-9. 36586066 PMC10202994

[27] Bai S. , Song D. , Chen M. , Lai X. , Xu J. , and Dong F. , The Association Between Mammographic Density and Breast Cancer Molecular Subtypes: A Systematic Review and Meta-Analysis, Clinical Radiology. (2023) 78, no. 8, 622–632, 10.1016/j.crad.2023.04.008, 37230842. 37230842

